# Excess Deaths and Hospital Admissions for COVID-19 Due to a Late Implementation of the Lockdown in Italy

**DOI:** 10.3390/ijerph17165644

**Published:** 2020-08-05

**Authors:** Raffaele Palladino, Jordy Bollon, Luca Ragazzoni, Francesco Barone-Adesi

**Affiliations:** 1 Department of Primary Care and Public Health, Imperial College, London W6 8RP, UK; 2 Department of Public Health, University “Federico II” of Naples, 80131 Naples, Italy; 3 CIRMIS—Interdepartmental Center for Research in Healthcare Management and Innovation in Healthcare, University “Federico II” of Naples, 80131 Naples, Italy; 4 Department of Translational Medicine, Università del Piemonte Orientale, 28100 Novara, Italy; jbollon94@gmail.com (J.B.); luca.ragazzoni@med.uniupo.it (L.R.); francesco.baroneadesi@uniupo.it (F.B.-A.); 5 CRIMEDIM—Research Center in Emergency and Disaster Medicine, Università del Piemonte Orientale, 28100 Novara, Italy

**Keywords:** COVID-19, lockdown, evaluation, late implementation, healthcare, health

## Abstract

In Italy, the COVID-19 epidemic curve started to flatten when the health system had already exceeded its capacity, raising concerns that the lockdown was indeed delayed. The aim of this study was to evaluate the health effects of late implementation of the lockdown in Italy. Using national data on the daily number of COVID-19 cases, we first estimated the effect of the lockdown, employing an interrupted time series analysis. Second, we evaluated the effect of an early lockdown on the trend of new cases, creating a counterfactual scenario where the intervention was implemented one week in advance. We then predicted the corresponding number of intensive care unit (ICU) admissions, non-ICU admissions, and deaths. Finally, we compared results under the actual and counterfactual scenarios. An early implementation of the lockdown would have avoided about 126,000 COVID-19 cases, 54,700 non-ICU admissions, 15,600 ICU admissions, and 12,800 deaths, corresponding to 60% (95%CI: 55% to 64%), 52% (95%CI: 46% to 57%), 48% (95%CI: 42% to 53%), and 44% (95%CI: 38% to 50%) reduction, respectively. We found that the late implementation of the lockdown in Italy was responsible for a substantial proportion of hospital admissions and deaths associated with the COVID-19 pandemic.

## 1. Introduction

In early January a novel strain of coronavirus, SARS-CoV-2, a virus which follows a human-to-human transmission, was identified in the Hubei province of China as the causative agent for a new disease later defined as Coronavirus Disease 2019 (COVID-19), a respiratory disease which is often characterized by influenza-like symptoms but which can also evolve (3–5% of the cases) into acute respiratory distress syndrome, or even sepsis, and multi-organ failure which might lead to death [1]. Starting from an outbreak in China, the scale of the emergency has rapidly grown globally, leading the World Health Organization (WHO) to declare the pandemic status on March 11th, 2020 when many countries had already introduced unprecedented physical distancing and containment measures to various extents [2]. As of May 28th, 2020 almost six million of COVID-19 cases and 361,836 deaths have been recorded worldwide [3].

The effect of containment measures in curbing the COVID-19 epidemic varied among countries [4,5,6,7,8,9]. While a combination of stringent policies together with wide early-phase testing coverage and effective contact tracing strategies was effective in halting the COVID-19 epidemic in countries such as mainland China, Hong Kong, and South Korea, in others the epidemic slowed only recently [3,5,6,8]. Factors explaining differences in time patterns might be found in the readiness of government responses and in the degree of compliance of the population to the implemented policies [5,6,7,8,9].

Italy, which has passed 232,000 confirmed cases and 33,000 deaths [10], is one of the most affected countries in the world so far and the first in Europe where the public health emergency rapidly escalated at the national level. On March 9th, 2020 the government ordered a national lockdown, a measure including: (a) strict home confinement of the entire population; (b) closure of all non-essential commercial activities; (c) mobility restrictions related to the involved municipalities [11]. The lockdown remained in place until May 3rd, when a slowdown of the epidemic in the different Italian regions allowed its release [12].

Compared with China, Italy introduced containment measures later in the course of the national epidemic, about one month after the first COVID-19 case was reported in the country. Italy’s lockdown was enforced 13 days after the one in Hubei, when normalizing for the time when the outbreak hit 50 cases in both countries [8]. This prompted a debate, in Italy and abroad, on the causes of such a delay and on how many COVID-19 cases could have been avoided, had the lockdown been implemented earlier [13]. A formal investigation into possible government mismanagement of the COVID-19 crisis is currently ongoing [14]. The aim of this study was to evaluate the health effects of late implementation of the lockdown in Italy. For this reason, we estimated the number of deaths and hospital admissions for COVID-19 that would have occurred if the lockdown had been implemented one week earlier than it was actually enforced.

## 2. Materials and Methods

In the present analysis we used data on the daily number of COVID-19 cases, hospitalized patients, and deaths recorded in Italy from February 24th, the first day national data were made available, to May 3rd, the last day of implementation of the national lockdown. Figures were provided by the official website of the Italian Department of Civil Protection [10].

First, we evaluated the effect of the Italian lockdown using interrupted time series (ITS) analysis. We modeled the time-series of daily new cases, *Y_t_*, using the following quasi-Poisson regression model, accounting for the possible overdispersion of data:

*log*(*Y_t_*) = *α* + *β*_1_*T* + *β*_2_*X_t_* + *β*_3_*T*_2_ + *e_t_*

where *T* is the time elapsed since the start of the study; *T*_2_ is the time elapsed since the implementation of lockdown (set to 0 before the lockdown); *X* is a dummy variable indicating the pre-lockdown period (coded 0) or the post-lockdown period (coded 1); *Y* is the logarithm of the number of new cases at time *T*; *α* is the intercept of the model; *β*_1_ represents the trend of new cases before the lockdown; *β*_2_ is the step change following the lockdown; *β*_3_ is the slope change following the lockdown; and *e_t_* is the error term of the model. Preliminary analysis of the data suggested that no adjustment was required for autocorrelation of the error terms *e_t_*. We also assumed a two-week lag between the implementation of the lockdown (March 9th) and the start of its effects (March 23rd), to take into account the COVID-19 incubation period and the diagnostic delay after symptoms onset [15].

Second, we evaluated the effect of an early lockdown on the trend of new cases, creating a counterfactual scenario where the lockdown was implemented one week in advance (i.e., on March 2nd instead of March 9th).

Third, based on the expected number of new cases, we predicted the corresponding number of intensive care unit (ICU) admissions, non-ICU admissions, and deaths, using a previously published mathematical model [16]. Briefly, the model simulates the progress of infected individuals between different compartments during the course of an epidemic: isolated at home, admitted in a non-ICU ward, admitted in ICU, recovered, dead. Finally, we compared the number of hospital admissions and deaths under the actual and counterfactual scenarios. All the analyses were performed using the R software (R Core Team (2013). R: A language and environment for statistical computing. R Foundation for Statistical Computing, Vienna, Austria. URL http://www.R-project.org/).

## 3. Results

**Figure 1:**
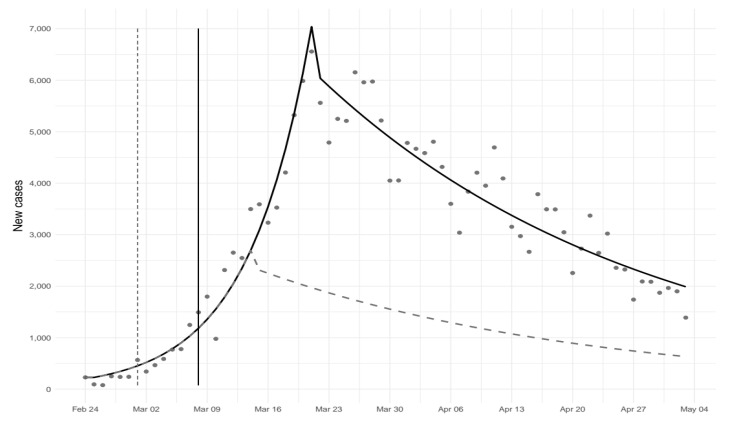
Predicted number of new cases of COVID-19 under different scenarios. Solid line represents the actual scenario (lockdown implemented on March 9th), and dashed line the counterfactual scenario (lockdown implemented on March 2nd).

From February 24th to May 3rd, 210,717 cases of COVID-19 were observed in Italy. There was an exponential increase in the number of new COVID-19 cases until March 22nd, followed by a sharp reduction (Table 1; Figure 1). Table 1 reports estimated coefficients, while related predictions are plotted in Figure 1 together with the expected number of new cases under the counterfactual scenario. On May 3rd, the number of new cases under the counterfactual scenario was less than half than that estimated under the observed scenario.

**Figure 2:**
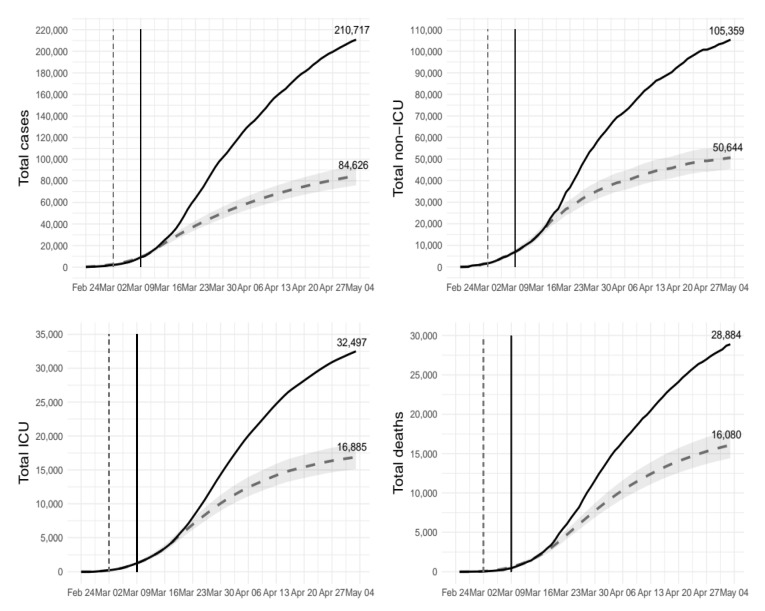
Total number of cases of COVID-19, non-ICU admissions, ICU admissions, and deaths under different scenarios. Solid line represents the actual scenario (lockdown implemented on March 9th) and dashed line the counterfactual scenario (lockdown implemented on March 2nd). ICU = intensive care unit.

Figure 2 shows differences in the total number of cases, non-ICU admissions, ICU admissions, and deaths under the two scenarios. The plots show that an early implementation of the lockdown would have averted about 126,000 COVID-19 cases, 54,700 non-ICU admissions, 15,600 ICU admissions, and 12,800 deaths. On the relative scale, this corresponds to a reduction of 60% (95%CI: 55% to 64%), 52% (95%CI: 46% to 57%), 48% (95%CI: 42% to 53%) and 44% (95%CI: 38% to 50%), respectively (Table 2).

Moreover, the maximum hospital demand would have been much lower under the counterfactual scenario. The peak number of non-ICU admissions would have been 14,336 rather than 29,010 (−51%; 95% CI: −45% to −56%). A similar reduction would be expected for ICU admissions as well (2300 vs. 4068 beds; −44%, 95%CI: −38% to −49%).

## 4. Discussion

In Italy, the COVID-19 pandemic led to the implementation of containment measures at the highest level, with a national lockdown enforced on March 9th, 2020. Despite this, by the time the epidemic curve started to flatten, the health system had already exceeded its capacity in different areas of the country, raising concerns that the public health response was indeed delayed. We found that if restrictive measures had been enforced one week earlier, this would have had a significant impact on the evolution of the epidemic in terms of hospital admissions and deaths. By May 3rd, we estimated that there would have been a 60% reduction of COVID-19 cases and 44% of confirmed deaths would have been averted.

The COVID-19 pandemic is threatening public health preparedness and medical response capacity globally. Our findings add to a growing body of evidence supporting the need for rapid responses to contain the current COVID-19 pandemic and similar threats that could occur in the future [5,6,12]. Besides Italy, other European countries profoundly impacted by the pandemic such as Spain, France, and the UK, as well as the US, also hesitated to enforce containment measures in a timely manner [8], with a consequent health, economic, and societal impact that still needs to be fully assessed. Lack of collaboration between national health systems, as well as delayed communication by international organizations might be some of the factors explaining the late response to the emergency. Public health intelligence at both the international and national level should identify all barriers and challenges associated with the current pandemic to improve response in the future. This is particularly necessary in this phase of the pandemic, as a possible second wave of infections is expected in the next months. As most European countries are gradually lifting restrictions, there is a need to enhance the existing surveillance systems and develop strategies for timely reactions to a new increase in the number of infections.

To our knowledge, this is the first study assessing the impact of the delay in the implementation of containment measures on the spread of COVID-19 epidemic, and the associated burden on the health system. However, several caveats merit discussion. First, analyses were conducted using publicly available data on confirmed cases, which did not account for the proportion of undetected cases, estimated to be high in Italy, especially in the regions more affected by the epidemic [17]. This means that, on the absolute scale, our estimates should be regarded as conservative. On the other hand, assuming that the timing of the lockdown is not associated with the detection rate, which seems plausible, the relative estimates provided are expected to be unbiased. Second, we did not take into account how the different hospital demand under the two scenarios affected the treatment of critical patients. In the actual scenario, hospitals in the worst-hit areas often exceeded their capacity and experienced ventilator shortages [8,16]. This affected their capacity to deliver effective care to all critical patients. On the other hand, under the counterfactual scenario the maximum hospital demand would have been about 50% lower. For this reason, we probably underestimated the positive effects of an early lockdown in terms of reduced ICU admissions and deaths.

## 5. Conclusions

The COVID-19 pandemic has been requiring unanticipated and extraordinary containment measures, which has raised concerns about public health preparedness of health systems globally. The late implementation of the lockdown in Italy was responsible for a substantial proportion of hospital admissions and deaths associated with the COVID-19 pandemic. Understanding factors contributing to such a delayed response is fundamental to strengthen public health preparedness and timing in response capacity.

## Figures and Tables

**Table 1:** Interrupted time series analysis. Estimated regression coefficients.

Coefficient	Estimate	95% CI	*p*-Value
α	5.29	(5.02 to 5.55)	<0.001
β1	0.14	(0.12 to 0.15)	<0.001
β2	−0.13	(−0.18 to −0.15)	0.062
β3	−0.16	(−0.18 to −0.15)	<0.001

**Table 2:** Changes in the number of cases of COVID-19, non-ICU admissions, ICU admissions, and deaths under the counterfactual scenario (lockdown implemented on March 2nd), compared to the actual scenario (lockdown implemented on March 9th). ICU = intensive care unit.

Study Outcome	Actual Scenario	Counterfactual Scenario	Relative Change (95% CI)
Total number of cases	210,717	84,626	−60% (−55% to −64%)
Total number of non-ICU admissions	105,359	50,644	−52% (−46% to −57%)
Total number of ICU admissions	32,497	16,885	−48% (−42% to −53%)
Total number of deaths	28,884	16,080	−44% (−38% to −50%)
Peak number of non-ICU admissions	29,010	14,336	−51% (−45% to −56%)
Peak number of ICU admissions	4068	2286	−44% (−38% to −49%)
